# Place of Residence and Cognitive Function in Older Adults in China: The Mediating Role of Social Participation

**DOI:** 10.3390/ijerph19010013

**Published:** 2021-12-21

**Authors:** Le Yang, Jingmin Cheng, Hongman Wang

**Affiliations:** 1 School of Management, Shanxi Medical University, 56 Xinjian South Road, Taiyuan 030001, China; jingmincheng@sxmu.edu.cn; 2 School of Health Humanities, Peking University, 38 Xueyuan Road, Beijing 100191, China; cde@pku.edu.cn

**Keywords:** social participation, older adults, place of residence, cognitive function

## Abstract

**Background:** Cognitive impairment is a severe health problem faced by older adults and their families, as well as the countries in which they live. Differences in place of residence may contribute to differences in the cognitive function of older adults, and the mediating effect of social participation has rarely been studied in China. **Methods:** A total of 10,014 older adult participants were included, using data from the Chinese Longitudinal Healthy Longevity Survey (CLHLS). Place of residence was described as either a city, town, or rural area. The frequency of participation in organized social activities and visits and interactions with friends was used to assess both formal and informal social participation. The Chinese version of a Mini-Mental State Examination (MMSE) was used as a measure of cognitive function. The mediation analysis was conducted using Hayes’ process version 3.4 on SPSS (IBM, Armonk, NY, USA). **Results:** Place of residence had a negative effect on cognitive function in older adults. The mediating functions of both informal (a1b1 = 0.199) and formal (a2b2 = −0.056) social participation indicate a suppression effect on the part of informal social participation and a partial mediation effect on the part of formal social participation in terms of the association between place of residence and cognitive function in older adults. Promoting both informal and formal social participation seems to be an important strategy for preventing a decline in the cognitive function of older adults, especially for those living in rural areas.

## 1. Introduction

Cognitive function is an important determinant of independence, daily living ability, and quality of life in older adults [1,2,3]. With increasing age, older adults tend to experience degenerative changes in their cognitive function in areas such as memory, attention, and executive functioning [4,5]. Recent research has shown that in China there are 15.07 million adults aged 60 years or older with dementia, and an additional 38.77 million older adults with mild cognitive impairment (MCI) [6]. Cognitive impairment will put great stress on the country, community, family and older adults themselves. As the overall population continues to age rapidly, these trends in cognitive impairment continue to increase. Given this, the issue of how to prevent cognitive decline and maintain cognitive function among older adults as well as how to provide social care for those with cognitive impairment has been the focus of researchers and experts on ageing, and a staple of the Healthy China Strategy and Healthy Ageing Strategy in China. Urban–rural differences remain a great challenge for China in its development, and the influence of urban–rural differences on the health of older adult has been getting more and more attention in academic research and policymaking.

### Literature Review

In order to formulate effective interventions for cognition impairment among older adults, it is essential to figure out its risk factors and mechanisms. Many studies have found that age, gender, income, marital status, education background, place of residence, smoking, drinking, physical activity, living environment, cognitive stimulation, and social relationships can all affect the cognitive function of older adults [7,8,9,10,11]. Among these factors, the association between place of residence and cognitive function in older adults has been researched in many previous studies [12,13,14], and the differences between urban and rural residence have been found to lead to variations in the cognitive function of older adults. Urban areas have convenient transportation as well as access to healthcare and health information, basic infrastructure, and educational opportunities, all of which improve the health and life quality of older adults living there. In contrast, older adults in rural areas need to face weak and inadequate health services [15], are often unattended if their children move to towns or cities for better education or work opportunities, and face other environmental and contextual obstacles [16], all of which contribute to older adults living in rural areas reporting worse health status, suffering more obesity, depression, and cognitive impairment, and being less socially active compared to those living in urban areas [17,18]. Jia J et al. (2014) found that in China, older adults in rural areas had a higher prevalence of dementia compared with their urban-dwelling counterparts [19] (Jia et al., 2014); the situation has been found to be similar in many other countries, such as Portugal [20] (Nunes et al., 2010) and Mexico [13].

Social participation or social engagement has often been used to explain the disparities in the health of older adults between urban and rural areas. When it comes to urban–rural differences in social participation, some studies have found that older adults living in urban areas have greater social participation, higher income, better transportation conditions, and more accessible community services, while the older adults living in rural areas were more likely to experience social exclusion and few contacts with friends [21,22,23,24]. Conversely, older adults living in rural places are often idealized as having stronger bonding social capital and possessing stronger ties to their communities [25], and a study from England has shown that older adults living in urban areas are more likely to experience social exclusion than older adults living in rural areas [26]. A better understanding of the residential characteristics that influence social participation is lacking. Levasseur et al. (2020) found that greater social participation was linked to an older population concentration in large metropolitan and rural areas, but not in urban areas [27]. In China, urban–rural differences should be researched from a hierarchical perspective; the differences between cities, towns, and rural areas show variations in social culture and geographical characteristics. Due to such trends as urbanization, refinement of the social division of labor, and changes in lifestyles, the interpersonal relationship with the environment in cities is more mobile and uncertain than in towns and rural areas, and contact between family members and friends may not be frequent enough, meaning that social participation may be affected. The traditional acquaintance relationships have gradually changed in cities, and social interaction patterns have changed as well. Compared with rural areas, life in towns is significantly more modern, convenient and rich; however, compared to cities, life in towns is more monotonous, simple, traditional, and backward. Additionally, the social participation status and pattern of older adults in rural areas may be changed as more and more young and middle-aged people migrate to towns or cities from rural areas.

Social participation has been proven to be beneficial to health, leading to improved mental and physical health, cessation of risky behaviors, and better-preserved cognitive function [28,29,30]. Given the increasing risks of ageing and high possibility of social isolation, the benefits brought by social participation play a strong role in the health of older adults. Social participation is defined as a person’s engagement in interactions with others [31,32]. Some studies have suggested that older adults are healthier when participating in more organizations, and that the influence of social participation on the health of older adults can vary depending on the different types of organizations. However, few studies consider the construct of social participation from the perspective of formal and informal social participation [33].

Although there is substantial evidence that both social participation and place of residence are associated with cognitive function among older adults, no study to date has systematically examined the differences in the association of place of residence and cognitive functions in older adults by city–town–rural status in China. Furthermore, few studies have addressed the potential for social participation as a mediator between place of residence and cognitive function based on the formal and informal social participation levels of older adults. Therefore, the primary objective of this study was to assess the association between place of residence and cognitive function in older adults in China. The secondary study objective was to explore the mediating role of social participation.

## 2. Methods

### 2.1. Study Design and Population

The Chinese Longitudinal Healthy Longevity Survey (CLHLS) was conducted on a randomly selected sample from half of the counties and cities in 23 of the 31 provinces in China, and is the largest set of survey data on older adults in China, covering about 85 percent of the total population [34,35,36]. To date, the CLHLS has conducted face-to-face interviews in 1998, 2000, 2002, 2005, 2008–2009, 2011–2012, 2014, and 2017–2018 using internationally compatible questionnaires conducted in the provinces’ respective native languages. Detailed information about the CLHLS including study design, sample distribution, data quality, and the contents of the data collected is available in previous studies [37,38].

There were 15,874 individuals involved in 2017–2018 (wave 8), with an average age of 85. The primary target population for the survey was persons aged 65 years and above. Respondents who did not answer all of the questions were excluded from the analysis. A final total of 10,014 responses were analyzed.

### 2.2. Measures

#### 2.2.1. Dependent Variables

Cognitive function was measured using the Chinese version of the Mini-Mental State Examination (MMSE), including orientation, short-memory, attention and calculation, recall, and language, with a score ranging from 0 to 30 [15,39]. The Chinese version of the MMSE, adapted from the standard version of the MMSE questionnaire developed by Folstein and his colleagues [40], reflects the cultural and socioeconomic conditions among older adults in China and uses questions that are easily understandable and practically answerable [41]. All MMSE questions in the CLHLS must be answered by the sampled person. The validity and reliability of the MMSE measures was tested and verified for each wave of the CLHLS [36,42]. Cognitive function as a continuous phenomenon with lower scores indicated higher cognitive impairment levels, with MMSE scores calculated as suggested by Zhang, Gu and Hayward (2010) [36]. A more detailed description of the Chinese version of the MMSE is available in previous research [43].

#### 2.2.2. Independent Variables

Place of residence was obtained using the question “Current residence area of interviewee” and was defined as an ordered categorical variable “city = 1, town = 2, and rural area = 3”. City refers to participants who lived in an urban core area or city district, town to those who lived in mixed urban and rural areas with urban communities concentrated as a bridge between urban and rural areas, and rural areas to those who lived in villages in the countryside or in more rural parts of cities and towns [44].

#### 2.2.3. Potential Mediating Variables

Mediating variables consisted of two types of social participation, formal and informal. Given previous studies [32,45], formal social participation was defined as involving attendance and social contact in preplanned activities, and informal social participation as involving more casual forms of social contact such as interaction with friends or out by oneself to engage socially.

Formal social participation was assessed by the frequency of participation in organized social activities. Informal social participation was assessed by the frequency of visits and interactions with friends. Responses ranged from 1 to 5, which meant “almost every day”, “at least once a week”, “at least once a month”, “sometimes”, and “never”.

#### 2.2.4. Covariates

This study selected the following as potential confounders: age, gender, year of education, marital status, and annual household income. Age, education (year of schooling), and annual household annual income were continuous variables. Marital status was grouped as “married and living with spouse, separate, divorced, widowed, never married”.

## 3. Data Analysis

First, descriptive statistics were calculated to summarize the respondents’ sociodemographic and other critical variables. Then, the effect of the independent variable on the dependent variable without the mediated variable was evaluated. This study performed multivariable multilevel linear regression analyses to explore the association between place of residence and cognitive function in older adults. Model 1 was crude, without any confounders, while Model 2 was adjusted for age, gender and education. In Model 3, as suggested by Zhang, Gu and Hayward (2010) [36] and Zeng et al. (2017) [38], we adjusted for marital status and household income based on the confounding variables in Model 2. Collinearity diagnostics were also conducted in linear regressions in order to assess the potential of regression coefficient instability. The tests for linear regression showed a VIF range from 1.139 to 1.529, which is lower than the recommended cut-off threshold of 10.

**Figure 1:**
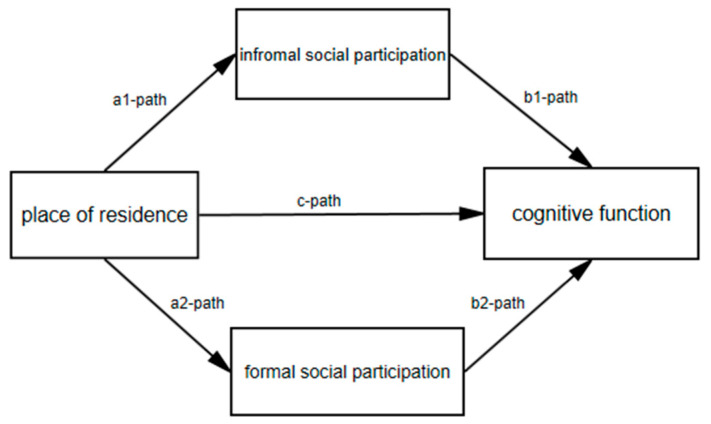
Overview of the analysis framework.

All covariates were tested as potential effect modifiers, but none of them turned out to be a significant effect modifier in the a- and b-paths, and were therefore included in the model as confounding variables. This paper explored the mediating role of social participation in the association between place of residence and cognitive function in older adults, adjusting for age, gender, education, marital status and household income. The proportion of the association between place of residence and cognitive function that was mediated by informal social participation and formal participation (path c’) was calculated by dividing ab by c. The association between place of residence and informal social participation (path a1) and formal social participation (path a2) was analyzed. Additionally, the associations between informal social participation and cognitive function (path b1) and between formal social participation and cognitive function (path b2) were explored. The mediating effects of informal social participation (a1b1) and formal social participation (a2b2) were calculated. Based on the above understanding, we proposed a theoretical framework (Figure 1).

All statistical analyses were performed using SPSS version 24.0 (IBM, Armonk NY, USA) along with Hayes’ process version 3.4 for SPSS. A *p* value < 0.05 was considered statistically significant. Bootstrapping was used to test the statistical significance of the direct, indirect, and total effects of the model [46,47].

## 4. Results

Table 1 presents the characteristics of the participants. More than half of the older adults were female (54.9%), the average age was 84.48 ± 11.8 years old, and the average amount of education was 3.87 ± 6.2 years. The average household annual income was RMB 46,113.7 ± 37,639.4, and 54.5% were widowed.

For the key variables, 41.0% lived in rural areas, and the mean score for MMSE (cognitive function) was 22.91 ± 9.0. Roughly one quarter of participants, 2554 (25.5%), regularly visited and interacted with friends, while 44.0% never did. With regard to formal participation, most of the respondents (85.0%) did not participate in organized social activities.

Table 2 showed the association between place of residence and cognitive function. In model 1 (crude model), a one-level increase in the place of residence was associated with a 1.067-point lower MMSE score (95% CI = −1.279; −0.855). Further adjustment for age, gender, education, marital status and household income weakened the association, and a one-level increase in the place of residence was associated with a 0.794-point lower MMSE score (95% CI = −0.988; −0.601).

Given the results presented in Table 3, place of residence showed a statistically significant association with informal social participation (path a1) and formal social participation (path a2). A one-level increase in place of residence was associated with a 0.221-point lower informal social participation (i.e., higher informal social participation frequency) and a 0.79-point higher formal social participation (i.e., lower formal social participation frequency).

Both informal social participation (path b1) and formal social participation (path b2) had positive effects on cognitive function. A one-point higher informal social participation was associated with a 0.901-point lower MMSE score (i.e., a lower informal social participation frequency was correlated with lower cognitive function) and a 0.319-point lower MMSE score (i.e., a lower formal social participation frequency was correlated with lower the cognitive function).

The mediation models revealed specific indirect effects. The specific indirect effects were a1b1 = 0.199 (through informal social participation) and a2b2 = −0.056 (through formal social participation). The total effect of place of residence on cognitive function in older adults as mediated by informal and formal participation was –0.788. The difference between informal social participation and formal social participation was statistically significant. The effect of place of residence on cognitive function in older adults was partially mediated by both informal and formal social participation. According to the results, the total effect was closer to zero than the direct effect, which indicates a suppression effect [48] from informal social participation.

## 5. Discussion

Using data from the CLHLS cross-sectional survey, social participation was found to play a mediating role in the association between place of residence and cognitive function in older adults, which indicates that differences in place of residence are associated with differences in social participation, and in turn have different influences on cognitive function in older adults. This study provided evidence for an association between different places of residence (city, town, and rural area) and cognitive function in older adults in China. It was found that place of residence had a negative effect on cognitive function, although the association was modest. Older adults living in rural areas showed a higher likelihood of a low MMSE score. As many previous studies in other countries have shown, older people living in urban areas have better cognitive function compared with their rural-dwelling counterparts [12,13,14,19]. The same consistently negative correlation between place of residence and cognitive function was found for older adults in China in this paper. Given the increasingly ageing population and functional differentiation of the different levels of administrative regions against the background of urbanization, smaller family structure, unequal medical resource allocation and multi-directional population migration, high importance has been attached to the consequences of and potential countermeasures to address inequality caused by differences in place of residence. Such policies as poor rural older adults in rural areas receiving full subsidies or exemptions from paying medical insurance fees, increasing pension insurance subsidies for rural residents, and strengthening health and old-age care for older adults in rural areas have achieved some results. However, improving the health and quality of life of older adults in their later years remains an important social issue in rural areas.

Social participation played a mediating role in the observed trends. Older adults living in rural areas had a higher frequency of informal social participation, and a lower frequency of formal social participation. Older adults with a higher frequency of informal and formal social participation had a lower risk of cognitive impairment, which could be due to the stress buffering effect of social participation. Social participation could provide a sense of belonging and enable older adults to better integrate into society through shared time and engagement in organized activities with friends and members of community groups, reducing their sense of solitude, buffering stress and leading to a more positive outlook on life [49,50]. Furthermore, social interaction could indirectly facilitate cognitive function in older adults by encouraging healthy behaviors [10] such as physical exercise, which has been proven to have an important positive impact on cognitive function in older adults [8,10,51,52].

The mediating effects of informal social participation and formal social participation were different. The results on the respective mediating effects indicated that informal social participation had a suppressing effect and formal social participation a partial mediating effect on the association between place of residence and cognitive function in older adults. This suppression can explain why a theoretically interesting relation is not very strong [53]. Place of residence was negatively associated with MMSE score among older adults, which showed that older people living in rural areas had worse cognitive function overall before considering informal social participation. However, when informal social participation was considered, the association was attenuated. It could be the case that rural-dwelling older people are more closely connected with communities, possess high-quality and stable neighborhood relations and friendships, and are more likely to have frequent interactions with neighbors and friends [25]. Due to population mobility, differences in work arrangements and lifestyle, and complete community functions and services, people living in cities have less neighborhood interaction and reciprocity; meanwhile, their friends may live far from their home, and the long drives and fast pace of life in cities can result in a lower frequency of informal social participation [24]. For formal social participation, the reason for the partial mediating effect may be the greater availability of senior-focused amenities, better transportation conditions, and diversity of social organizations and groups which cities provide to older adults living there, with greater opportunity to participate in organized social activities and a more open and inclusive culture which increases the willingness of older people to participate.

In addition, more attention needs to be paid to older adults who lose their family or friends that have died or moved away, or who relocate or follow their children to the city; they may not be able to integrate into the new living environment when their original social connections are weakened. Rural-dwelling older adults in China engage in more informal social participation and less formal participation, which can place them in disadvantageous situations. Policymakers should encourage and advocate participation by older adults living in rural areas in more organizations and organized social activities in order to expand their social networks and strengthen their connections to outside sources of support so as to effectively utilize the protective effects of social participation on the cognitive functioning of older adults.

This study had three limitations. First, our findings are based on cross-sectional data; thus, no causal relationships can be established. Second, there are different types of social participation, and their mediating effects may be varied. This paper does not distinguish types of social participation or capture precise varieties of informal social participation or formal social participation due to a lack of data. Without such information, the accuracy of our estimation of the mediating effect of social participation may be compromised. Future studies should explore potential differences in the mediating effects of different types of social participation. Lastly, although this paper revealed the mediating effect of social participation in the association between place of residence and cognitive function, the underlying mechanisms remain unclear and notional, and will be the focus of our future research.

## 6. Conclusions

This study found that social participation plays a mediating role in the association between place of residence and cognitive function in older adults in China. Informal social participation has a suppressing effect, while formal social participation has a partial mediating effect. These findings could provide evidence and inspiration for health interventions for older adults in China. Differences in place of residence affect the health of older people in different ways, such as access to resources and living environments; informal and formal social participation could be used to attenuate these differences and their respective influences. In follow-up research, the mechanisms involved in the mediating role of social participation need to be further explored using longitudinal data.

## Figures and Tables

**Table 1:** Characteristics of the older adults (*n* = 10,014).

	*n* (%)	M (SD)
Gender		
Male	4516 (45.1%)	
Female	5498 (54.9%)	
Age		84.48 (11.8)
Household annual income		46,113.7 (37,639.4)
Education		3.87 (6.2)
Marital status		
Married and living with spouse	4296 (42.9%)	
Separated	150 (1.5%)	
Divorced	40 (0.4%)	
Widowed	5458 (54.5%)	
Never married	80 (0.8%)	
Current residential area		
City	2724 (27.2%)	
Town	3184 (31.8%)	
Rural	4106 (41.0%)	
Informal social participation		
almost every day	2554 (25.5%)	
at least once a week	1542 (15.4%)	
at least once a month	661 (6.6%)	
sometimes	841 (8.4%)	
never	4406 (44.0%)	
Formal social participation		
almost every day	300 (3.0%)	
at least once a week	300 (3.0%)	
at least once a month	320 (3.2%)	
sometimes	571 (5.7%)	
never	8512 (85.0%)	
MMSE score		22.91 (9.0)

**Table 2:** Multiple linear regression coefficients of the association between place of residence and cognitive function (N = 10,014).

	Model 1	Model 2	Model 3
Place of residence	−1.067 *** (−1.279; −0.855)	−1.010 *** (−1.189; −0.830)	−0.794 *** (−0.988; −0.601)

Model 1: crude model. Model 2: adjusted for age, gender, education. Model 3: adjusted for age, gender, education, marital status, and household income. *** *p* < 0.001. B = coefficient, 95% CI = 95% confidence interval.

**Table 3:** Mediation of effect of place of residence on cognitive function through informal social participation and formal participation. ISP: informal social participation; FSP: formal social participation.

	Path a (B, 95% CI)	Path b (B, 95% CI)	Path c (B, 95% CI)	Path c’ (B, 95% CI)	Different Comparison
ISP	−0.221 ** (−0.263; −0.179)	−0.901 ** (−0.990; −0.811)	−0.932 ** (−1.125; −0.738)	0.199 ** (0.159; 0.243)	0.255 ** (0.211; 0.302)
FSP	0.174 ** (0.150; 0.197)	−0.319 ** (−0.482; −0.158)		−0.056 ** (−0.078; −0.034)	

Path a represents the association between the place of residence and ISP/FSP. Path b represents the association between ISP/FSP and cognitive function. Path c represents the direct association between the place of residence and cognitive function. Path c’ represents the indirect association between the place of residence and cognitive function. Different comparison represents the specific indirect contrast of ISP and FSP (i.e., a1b1-a2b2). Associations are adjusted for age, gender, education, marital status, and household income. Standardized estimation of 5000 bootstrap samples, ** *p* < 0.05. B = coefficient, 95% CI = 95% confidence interval.

## Data Availability

PKU Centre for Healthy Ageing and Development. 2021. “Chinese Longitudinal Healthy Longevity Survey (CLHLS).” Peking University Open Research Data. https://doi.org/10.18170/DVN/WBO7LK (accessed on 16 December 2021).
